# Forecasting the rate of hand injuries in Singapore

**DOI:** 10.1186/s12995-022-00350-6

**Published:** 2022-05-04

**Authors:** Liau Zi Qiang Glen, Joel Yat Seng Wong, Wei Xuan Tay, Jiayi Weng, Gregory Cox, Andre Eu Jin Cheah

**Affiliations:** 1 Department of Orthopaedic Surgery, National University Health System, Singapore, Singapore; 2 Department of Orthopaedic Surgery, NUHS Tower Block, Level 11, 1E Kent Ridge Road, Singapore, 119288 Singapore; 3 University Orthopaedic, Hand and Reconstructive Microsurgery Cluster, National University Health System, 5 Lower Kent Ridge Rd, Main Building 1, Level 2, Singapore, 119074 Singapore; 4 Department of Economics, National University of Singapore, 1 Arts Link, Singapore, 117568 Singapore; 5 Department of Hand & Reconstructive Microsurgery, National University Hospital, 5 Lower Kent Ridge Rd, Main Building 1, Level 2, Singapore, 119074 Singapore

**Keywords:** Demand, Supply, Labour, Health, J2, I1

## Abstract

**Purpose:**

This study aims to analyse the correlation between the incidence rate of hand injuries and various major economic indicators in Singapore. We hypothesise that the number of hand injuries is correlated to activity in the construction and manufacturing industries in Singapore.

**Methods:**

Twenty thousand seven hundred sixty-four patients who underwent hand surgeries in a tertiary institution between 2012 to 2018 were reviewed. Two independent, blinded observers extracted the frequency of hand surgeries performed from Electronic Medical Records. Economic indicators pertinent to Singapore’s economic activity were collected and smoothed by simple moving average of the prior 3 months. Results were analysed using IBM SPSS v25.0.

**Results:**

Significant independent univariate variables were Purchasing-Manager-Index and Industrial-Production-Index. Multiple linear regression of quarterly reported figures showed that Total-Livestock-Slaughtered, Total-Seafood-Handled, Purchasing-Manger-Index, Industrial-Production-Index, Gas-Tariffs, Construction-Index, Consumer-Price-Index, Total-Air-Cargo-Handled, Total-Container-Throughput, Total-Road-Traffic-Accident-Casualties, Food-&-Beverage-Services-Index were significantly correlated (*p* < 0.05) with hand injuries, with *R*^2^ = 62.3%.

**Conclusion:**

Quarterly economic indicators from major economic industries can be used to predict the incidence of hand injuries with a 62.3% correlation. These findings may be useful for anticipating healthcare resource allocation to treat hand injuries.

**Type of study and level of evidence:**

Economic and decision, Level II.

## Introduction

Hand injuries are frequently encountered by doctors involved in trauma care [1]. Studies in the Emergency Department (ED) have shown that hand injuries comprise up to 10% of the visits and range from simple lacerations or finger sprains to mutilating injuries or amputations [2]. The high incidence rate of hand injuries in Singapore is not limited to those that present to emergency departments. AIG Asia Pacific Insurance Pte. Ltd. analysed 3500 claims in a 12-month period and found that hand injuries accounted for 36% of the overall claims reported. In contrast, slip, trip and fall accidents contributed 24% [3]. Hand injuries make up a significant percentage of acute injuries in Singapore and form the most common traumatic occupational injury [4].

Various studies have evaluated the epidemiology of wrist and hand injury in numerous countries. Burridge JD et al. reported that the occupations in New Zealand with the highest rate of hand injuries are machine operators, construction, forestry workers and meat processing workers. Michal Grivna et al. [5] found that hand injuries constitute 7–28% of all injuries in the United Arab Emirates (UAE) [6, 7], and account for approximately 20% of all emergencies presenting to hospital emergency departments [8]. Polinder S. et al. found that in the Netherlands, 42% of all emergency department visits were due to upper extremity injuries, of which injuries to the wrist and hand were the most common [9].

Construction and manufacturing industries are the highest contributors of hand injuries. AIG Asia Pacific Insurance Pte. Ltd. reports that 35% of the total annual claims value was incurred by workers in the construction and manufacturing trade [3]. Hey et al. studied 504 patients admitted to the Emergency Department of National University Hospital (NUH) Singapore with hand injuries over a course of 3 months and found that 43% of these patients suffered from industrial injuries, comprising of lacerations and crush trauma [10]. If there is a significant correlation between hand injuries and activity in the construction and manufacturing industries, a fluctuation in the labour force of these industries may cause the number of hand injuries to fluctuate correspondingly. Singapore’s labour workforce is flexible, with the total labour force fluctuating over the years. The Report on Labour Force in Singapore by the Ministry of Manpower details that between 1994 to 1996, the percentage change was 4.5 to 5.04% to 15.75% respectively; and from 2000 to 2002, the percentage change was − 0.74 to 6.3% to − 0.42% respectively [11]. Moreover, as Singapore has long counted on its people as its biggest resource, Singapore’s economy is heavily dependent on foreign labour, particularly in blue collar jobs. There are 1,382,900 foreign workers (36% of Singapore’s total labour force), of which approximately 300,000 of them are in the construction industry [12], while 50% of the manufacturing industry are foreign workers [13].

Workers in sectors requiring manual labour, in particular the construction and manufacturing industry, tend to face a higher risk of suffering from hand injuries. Grivna et al. found that in the UAE, workers in the construction industry were at a higher risk of suffering hand injuries. Hand injury rates of workers operating machinery was 51.1% compared to heavy objects (27.7%), traffic (0%) and animal (0%) [5]. In Singapore, other labour-intensive industries such as transport and agriculture have also been reported to contribute to the burden of hand injuries [14, 15].

To better inform public health strategies to combat the prevalence of hand injuries, it is important to understand how various economic factors might be associated with hand injuries. Mathematical and statistical models can provide substantial contributions to the understanding of growth trends in the incidence of hand injuries. Machine learning has been proposed as an appealing approach for building models with more predictive power than linear regressions [16]. Among these models, the seasonal autoregressive integrated moving average (SARIMA) model is useful in situations when the time series data exhibit seasonality-periodic fluctuations that recur with about the same intensity each year [17] and its use in forecasting time series models have been widely reported [17–22]. This characteristic makes the SARIMA model adequate for studies concerning monthly data of the incidence of hand injuries, given that the number of hand injuries tends to be subject to seasonal variations in economic activity. However, the trade-off of most machine learning models is that they are based on mathematical functions that do not have readily interpretable coefficients [16] in contrast to linear regressions. Hence, the use of linear regression to identify factors associated with a particular outcome has been reported by various sources [16, 22–25].

To the author’s knowledge, no prior studies have been conducted to evaluate the correlation between the incidence rate of hand injuries and manpower in the manufacturing, construction, transport and agriculture industries in Singapore.

Our study aims to analyse the correlation between the incidence rate of hand injuries and manpower in major economic industries such as the manufacturing, construction, transport and agriculture industries in Singapore. Our objectives were to determine which factors best explain variation in hand injuries and whether traditional linear regression methods or SARIMA models are best suited for doing so. We hypothesise that economic indicators in the manufacturing, construction, transport and agricultural industries are directly correlated to the rate of hand injuries.

## Methods

### Data sources

Twenty thousand, seven hundred and sixty-four consecutive patients who had undergone Hand & Reconstructive Microsurgery (HRM) surgeries in a single tertiary trauma institution between January 2012 and December 2018 were included in our retrospective study. A non-observer team member extracted the relevant patient data from the Electronic Medical Records and performed de-identification of data. The study was performed by two independent observers who were blinded to the patient identifiers.

Various indicators pertinent to Singapore’s economy were collected from online published data by Singapore government agencies. The Singapore Standard Industrial Classification (SSIC) 2020 was used to ensure all major economic industries were accounted for. SSIC is the national standard for classifying economic activities undertaken by economic units and is used in censuses of population, household and establishment surveys and in administrative databases. The SSIC adopts the basic framework and principles of the International Standard Industrial Classification of All Economic Activities (ISIC). It is reviewed and updated regularly to reflect significant changes in the structure of the Singapore economy and the emergence of new activities as well as to align with changes in international standards [26]. Details of the economic markers used and their corresponding abbreviations appear in Table 1. Some economic markers include Total Livestock Slaughtered (AgriLive) and Total Seafood Handled (AgriFish) to represent the Agriculture and Fishing classification; Purchasing Manager Index (PMI) and Industrial Production Index (IPI) to represent Manufacturing; Gas Tariffs (Gas) to represent Gas Supply; Construction Index (CI) to represent Construction; Consumer Price Index (CPI) to represent Wholesale and Retail Trade; Total Air Cargo Handled (Loaded and Discharged) (TransAir) and Total Container Throughput (TransSea) to represent Transportation and Storage; and Food & Beverage Services Index (F&B) to represent Food Service Activities.

**Table 1:** List of economic markers relevant to Singapore’s major economic industries, according to the Singapore Standard Industrial Classification (SSIC) 2020 classification

	Classification [26]	Economic Marker	Abbreviation	Unit of Measurement	Source	Frequency	Reason for inclusion
A	Agriculture and Fishing	Total Livestock Slaughtered	AgriLive	Number	Department of Statistics Singapore	Monthly	Reflects the level of agricultural livestock activity
		Total Seafood Handled	AgriFish	Tonnes	Department of Statistics Singapore	Monthly	Reflects the level of agricultural fishing activity
B	Manufacturing	Manufacturing Index	MI	Points	CEIC Data, Department of Statistics	Monthly	Reflects manufacturing activity in Singapore
		Purchasing Manager Index	PMI	Percentage	Singapore Institute of Purchasing and Material Management	Monthly	Surveys purchasing managers at businesses in the Manufacturing sector, is a key barometer of the Singapore manufacturing economy
		Industrial Production Index	IPI	Long-term average (hundreds)	Singapore Economic Development Board	Monthly	Real production output of manufacturing, mining, and utilities.
C	Electricity, Gas, Steam and Air-conditioning Supply	Electricity Generation and Consumption	ElectGenCon	Gigawatt Hours	Department of Statistics, Singapore	Monthly	Reflects the amount of electricity generated and consumed
		Gas Tariffs	Gas	Cent Per Kilowatt Hour	Department of Statistics, Singapore	Monthly	Reflects the economic activity of gas
D	Water Supply; Sewerage, Waste Management and Remediation Activities	Water Sales	WaterS	Million Cubic Metres Per Year	Department of Statistics, Singapore	Annual	Reflects the economic acitivty of water supply
E	Construction	Construction Index	CI	Points	CEIC Data, Department of Statistics	Monthly	Reflects the level of construction activity in Singapore
F	Wholesale and Retail Trade	Consumer Price Index	CPI	Price	Department of Statistics Singapore	Monthly	Measures the average price changes over time of a fixed basket of consumption goods and services commonly purchased by the resident households.
G	Transportation and Storage	Total Air Cargo Handled (Loaded and Discharged)	TransAir	Number	Department of Statistics Singapore	Monthly	Reflects the level of air transport
		Total Container Throughput	TransSea	Thousand Twenty-Foot Equivalent Units (‘000 TEUs)	Department of Statistics Singapore	Monthly	Reflects the level of sea transport
		Total Road Traffic Accident Casualties	TransLand	Number	Department of Statistics Singapore	Monthly	Reflects the level of land transport
H	Accommodation and Food Service Activities	Residential Units Constructed and Sold by Housing and Development Board	ResUnits	Number	Department of Statistics Singapore	Annual	Reflects the demand and supply of public housing
		Food & Beverage Services Index	F&B	Index	Department of Statistics Singapore	Monthly	Reflects the demand and supply of food and beverage industries
I	Financial and Insurance Activities	Turnover On The Singapore Exchange (Equities) - Mainboard (Volume)	SGX	Million	Department of Statistics Singapore	Monthly	Reflects the level of financial activity in Singapore
J	Administrative and Support Service Activities	Government Operating Revenue	GOR	Million	Department of Statistics Singapore	Monthly	Reflects the level of government indication
K	Public Administration and Defence	Total Fire Calls	Fire	Number	Department of Statistics Singapore	Annual	Reflects the activity of the firefighting services
		Crime Cases Recorded	Police	Number	Department of Statistics Singapore	Annual	Reflects the activity of the police services
L	Education	Enrolment In Educational Institutions	Edu	Number	Department of Statistics Singapore	Annual	Reflects the demand of education in Singapore
M	Health and Social Services	Admissions To Public Sector Hospitals	HealthC	Number	Department of Statistics Singapore	Monthly	Reflects the demand of healthcare in Singapore
N	Arts, Entertainment and Recreation	Utilisation Of Sports Facilities	Sports	Total Bookings (Number)	Department of Statistics Singapore	Annual	Reflects the recreational physical activity of people residing in Singapore
		Tickted Attendances in the Performing Arts	Arts	Number	Department of Statistics Singapore	Annual	Reflects the level of The Arts in Singapore

Since Singapore lacks both arable land and natural resources pertaining to fuels, metals or minerals [27, 28], mining activities are negligible [29]. Hence mining activities were not included in our analysis. Agricultural activity in Singapore consists of poultry and fishing farms [27, 28, 30].

### Subgroup analysis

To obtain a better understanding of how demographics could affect forecasting results, the 20,764 patients were segmented into subgroups of age (17 to 62 years old), citizenship status (Singaporean vs. non-Singaporean) and gender (male vs. female). Analysis was conducted on these subgroups. Seventeen to sixty-two years old was chosen since the legal age to work in Singapore is 17 years and above [31, 32], while the minimum retirement age is 62 years. The expectation was that those within the legal working ages may have increased risk of sustaining hand injuries.

### Statistical analysis

IBM SPSS software version 25.0 was used for data analysis. Chi Square test was performed to compare the various economic indicators against incidence rate of hand injuries. R-square (R^2^), a measure of how well the model fits the data (‘goodness of fit’) [33] and *p*-value were calculated for all subgroup analyses. Statistical significance was defined as *p*-value < 0.05. This study was approved by an Institutional Review Board.

#### Linear regression model

Univariate linear regression models were used to determine the association between hand injuries and each economic indicator. Multiple linear regression model was used to find the association between hand injuries and variables of the major economic groups. We conducted a sensitivity analysis at the one-, three-, six-, nine- month and yearly interval and found that calculations using simple moving average of the prior 3 months resulted in the best forecasting results. An equation was formulated using multiple linear regression to predict the incidence of hand injuries.

#### SARIMA model

**Fig. 1:**
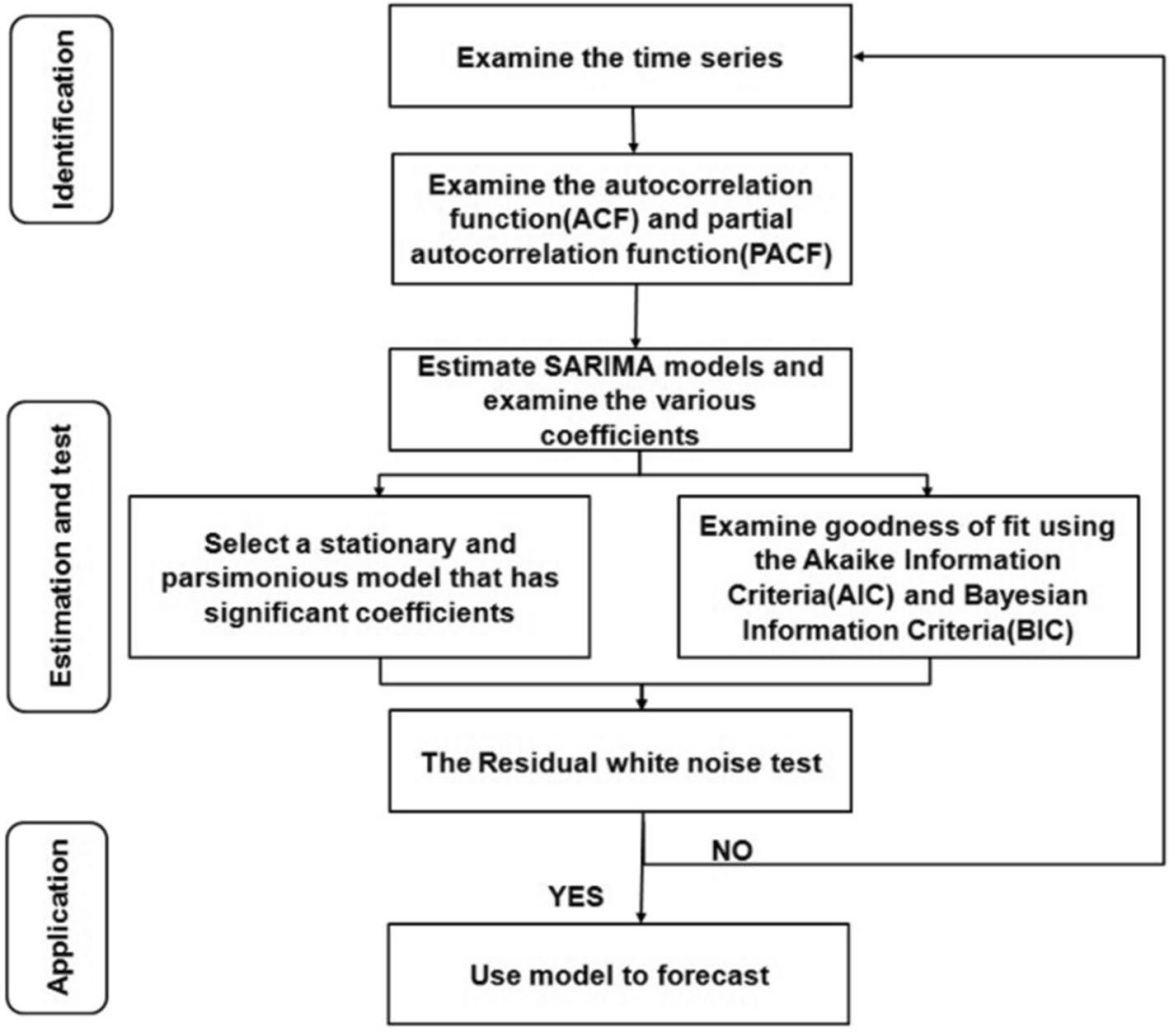
The process and method of seasonal autoregressive integrated moving average (SARIMA) model [21]

The data on hand injuries from 2012 to 2018 was used as the forecasting dataset to derive an R^2^ value. We established and selected the best SARIMA model (p, d, q) × (P, D, Q) according to the steps introduced by Box and Jenkins [21, 34] (Fig. 1), including minimizing the Bayesian information criterion (BIC). Autoregressive lags, moving average lags, seasonal autoregressive lags, and seasonal moving average lags are indicated by p, q, P, and Q, respectively [35].

### Comparison of multiple linear regression model and SARIMA model

The R^2^ of the multiple linear regression in the monthly and quarterly subgroup analyses was used to compare against the R^2^ of SARIMA to determine the most accurate multiple linear regression model.

## Results

### Demographics

73.68% of our patients were between 21 to 60 years old and 82.17% were between the legal working ages of 17 to 62 years old [31, 32]. There was a male predominance (71.57%), 53.69% of our patients were Chinese and 58.79% were Singaporean residents (Table 2).

**Table 2:** Demographics of 20, 764 patients according to age, gender, nationality and race

	Percentage (%)
**Age (years old)**^a^	
1 to 20	9.77
21 to 40	42.13
41 to 60	31.55
61 to 80	15.44
80 to 98^b^	1.11
17 to 62^c^	82.17
**Gender**	
Male	71.57
Female	28.43
**Nationality**	
Singaporean	58.79
Non-Singaporean	41.21
**Race**^d^	
Chinese	53.68
Malay	11.59
Indian	15.58
Others	19.14

^a^Mean age is 40.93 years old

^b^Oldest patient is 98 years old

^c^Legal age to work in Singapore is 17 years and above [31, 32], while the minimum retirement age is 62

^d^The “Chinese, Malay, Indian, Others (CMIO) model” is the dominant organizing framework of race in Singapore [36, 37]

### SARIMA model

For monthly analysis, the model with the lowest BIC value, and therefore the best-fit model, was SARIMA (0,1,1)(0,1,1)_12_ yielding an R^2^ value of 0.238. SARIMA (0,1,1)(0,1,1)_12_ with R ^2^ value of 0.747 was the best-fit model for the quarterly analysis.

### Linear regression

The results for the univariate analysis and multiple linear regression analysis conducted via SPSS to analyse the correlation between the various economic variables and the incidence rate of hand injuries are shown in Tables 3, 4 and 5.

**Table 3:** Linear regression for the Main^a^ and Citizenship subgroup

	Main^**a**^		SG		nonSG	
	R^**2**^	***p*** value	R^**2**^	***p*** value	R^**2**^	***p*** value
**Monthly**						
**AgriLive**	0.002	0.706	0.031	0.112	0.103	0.003*
**AgriFish**	< 0.001	0.943	0.019	0.209	0.099	0.004*
**MI**	0.003	0.637	0.079	0.010*	0.305	< 0.001*
**PMI**	0.084	0.008*	0.112	0.002*	0.016	0.257
**IPI**	0.053	0.035*	0.115	0.002*	0.075	0.012*
**ElecGenCon**	0.141	< 0.001*	0.314	< 0.001*	0.217	< 0.001*
**Gas**	0.001	0.763	0.098	0.004*	0.455	< 0.001*
**CI**	0.009	0.390	0.063	0.022	0.143	< 0.001*
**TransAir**	0.036	0.085	0.138	0.001*	0.200	< 0.001*
**TransSea**	0.121	0.001*	0.118	0.001*	0.000	0.922
**TransLand**	0.010	0.374	0.028	0.129	0.029	0.122
**F&B**	0.005	0.544	0.003	0.640	0.079	0.009*
**SGX**	0.018	0.230	0.096	0.004*	0.187	< 0.001*
**GOR**	0.041	0.066	0.111	0.002*	0.329	0.002*
**HealthC**	0.054	0.033*	0.212	< 0.001*	0.312	< 0.001*
**Quarterly**						
**AgriLive**	0.008	0.660	0.039	0.317	0.369	0.001*
**AgriFish**	0.051	0.247	0.219	0.012*	0.311	0.002*
**MI**	0.004	0.757	0.148	0.044*	0.508	< 0.001*
**PMI**	0.161	0.034*	0.196	0.018*	0.020	0.471
**IPI**	0.132	0.057	0.222	0.011*	0.078	0.149
**ElecGenCon**	0.030	0.381	< 0.001	0.986	0.131	0.058
**Gas**	0.066	0.187	0.004	0.750	0.152	0.040*
**CI**	0.020	0.471	0.100	0.101	0.159	0.036*
**CPI**	0.069	0.178	0.169	0.030*	0.130	0.060
**TransAir**	0.049	0.259	0.226	0.011*	0.341	0.001*
**TransSea**	0.187	0.021*	0.162	0.034*	0.000	0.951
**TransLand**	0.001	0.853	0.007	0.663	0.069	0.175
**F&B**	< 0.001	0.939	0.151	0.041*	0.774	< 0.001*
**SGX**	0.001	< 0.001*	0.029	0.384	0.104	0.095
**GOR**	0.119	0.073	0.036	0.334	0.087	0.128
**HealthC**	0.011	0.599	0.031	0.369	0.359	0.001

*SG* Singaporean, *nonSG* non-Singaporean

^a^Refers to analysis of the entire population of hand injuries without the subgroup analysis

*Statistically significant (*p* < 0.05)

**Table 4:** Linear regression for the Gender and Age^a^ subgroups

	Gender (Male)		Gender (Female)		Age^**a**^	
	R^**2**^	***p*** value	R^**2**^	***p*** value	R^**2**^	***p*** value
**Monthly**						
**AgriLive**	0.020	0.204	0.045	0.052	0.042	0.062
**AgriFish**	0.128	0.001*	0.138	0.001*	0.064	0.021*
**MI**	0.066	0.019*	0.039	0.072	0.092	0.005*
**PMI**	0.017	0.231	0.103	0.003*	0.001	0.734
**IPI**	0.011	0.343	0.037	0.079	0.007	0.449
**ElecGenCon**	0.021	0.188	0.158	< 0.001*	0.004	0.579
**Gas**	0.173	< 0.001*	0.213	< 0.001*	0.136	0.001*
**CI**	0.171	< 0.001*	0.531	< 0.001*	0.056	0.031*
**TransAir**	0.024	0.156	0.048	0.046*	0.024	0.163
**TransSea**	0.003	0.650	0.028	0.129	0.014	0.276
**TransLand**	0.034	0.095	0.002	0.685	0.004	0.572
**F&B**	0.002	0.693	0.053	0.034*	0.006	0.501
**SGX**	0.001	0.762	0.028	0.126	0.014	0.285
**GOR**	0.013	0.297	0.033	0.097	< 0.001	0.872
**HealthC**	0.065	0.019*	0.151	< 0.001*	0.050	0.040*
**Quarterly**						
**AgriLive**	0.177	0.026*	0.109	0.086	0.314	0.002*
**AgriFish**	0.157	0.037*	0.297	0.003*	0.085	0.133
**MI**	0.101	0.099	0.053	0.237	0.186	0.022*
**PMI**	0.032	0.361	0.120	0.070	0.006	0.687
**IPI**	0.008	0.658	0.047	0.270	0.011	0.591
**ElecGenCon**	0.062	0.200	0.004	0.750	0.126	0.064
**Gas**	0.322	0.002*	0.093	0.114	0.337	< 0.001*
**CI**	0.204	0.016*	0.516	< 0.001*	0.076	0.155
**CPI**	0.034	0.344	0.065	0.190	0.043	0.287
**TransAir**	0.066	0.186	0.071	0.170	0.093	0.114
**TransSea**	< 0.001	0.970	0.037	0.327	0.004	0.736
**TransLand**	0.004	0.756	0.011	0.599	0.003	0.778
**F&B**	0.261	0.006*	0.259	.006*	0.353	0.001*
**SGX**	0.004	0.757	0.016	0.525	< 0.001	0.922
**GOR**	0.256	0.006*	0.082	0.139	0.285	0.003*
**HealthC**	0.255	0.006*	0.127	0.062	0.312	0.002*

^a^17 to 62 years of age

*Statistically significant (*p* < 0.05)

**Table 5:** Multiple linear regression

	AgriLive	AgriFish	MI	PMI	IPI	ElecGenCon	Gas	CI	CPI	TransAir	TransSea	TransLand	F&B	SGX	GOR	HealthC	(Constant)	R^**2**^	***p*** value
	Coefficients																		
**Monthly**																			
**SARIMA**	NA	NA	NA	NA	NA	NA	NA	NA	NA	NA	NA	NA	NA	NA	NA	NA	NA	0.238	NA
**Main**^**a**^	-1.853E-5*	−0.003*	2.356*	8.225*	0.155*	0.059*	−4.732*	−0.544*	NA	−0.001*	−0.022*	0.039*	−0.275*	−3.870E-4*	0.002*	0.009*	− 1325.640	0.225	0.004*
**Age**^**b**^	−2.846E-5*	−0.002*	2.748*	3.315*	−0.078*	0.045*	−7.237*	−1.015*	NA	−0.001*	−0.012*	0.038*	−0.224*	−0.001*	0.002*	0.008*	− 959.874	0.275	0.001*
**SG**	−1.178E-5*	−0.003*	1.890*	7.401*	0.254*	0.055*	−2.833*	−0.285*	NA	−0.358E-4*	−0.040*	0.040*	−0.590*	< 0.001*	0.003*	0.009*	− 1197.257	0.332	< 0.001*
**nonSG**	< 0.001*	< 0.001*	0.466*	0.823*	−0.099*	0.004*	−1.899*	−0.259*	NA	< 0.001*	0.018*	< 0.001*	0.314*	< 0.001*	− 0.002*	0.001*	−128.383	0.486	< 0.001*
**Gender (M)**	−2.2E-05*	0.000413*	2.121*	3.986*	−0.138*	0.048*	−1.202*	−1.117*	NA	−0.001*	−0.043*	0.117*	−1.364*	−0.001*	< 0.001*	0.007*	−589.988	0.409	< 0.001*
**Gender (F)**	−2.3E-05*	0.001985*	1.742*	−13.038*	−0.180*	0.035*	−8.798*	−2.401*	NA	< 0.001*	−0.042*	0.015*	−2.179*	−0.002*	0.001*	< 0.001*	1436.240	0.839	< 0.001*
**Quarterly**																			
**SARIMA**	NA	NA	NA	NA	NA	NA	NA	NA	NA	NA	NA	NA	NA	NA	NA	NA	NA	0.747	NA
**Main**^**a**^	−4.183E-5*	−0.003*	NS	11.640	2.574*	NS	− 0.034*	0.908*	−24.749*	0.001*	NS	0.145*	−7.372*	NS	NS	NS	2736.439	0.623	0.030*
**Age**^**b**^	−5.654E-5*	−0.003*	0.506*	NS	2.360*	1.463*	−0.072*	0.130*	−21.801*	0.001*	0.011*	0.160*	−7.334*	< 0.001*	NS	−0.008*	5137.978	0.477	0.038*
**SG**	−3.099E-5*	−2.566*	NS	NS	2.590*	1.168*	−0.184*	2.359*	−15.415*	0.001*	0.068*	NS	−9.984*	< 0.001*	0.026*	NS	6047.327	0.430	0.036*
**nonSG**	−1.905E-5*	5.777E-5*	0.521*	4.210*	0.026*	0.034*	0.040*	−0.384*	−4.674*	1.99E-4*	0.021*	0.067*	−1.211*	−1.27E-4*	−0.006*	−0.002*	− 12.579	0.898	< 0.001*
**Gender (M)**	−3.534E-5*	0.005*	−3.232*	−14.370*	3.151*	1.138*	−0.150*	NS	−24.514*	< 0.001*	0.044*	0.080*	−7.779*	< 0.001*	NS	−0.011*	10,420.887	0.461	0.044*
**Gender (F)**	−4.579E-5*	0.010*	−5.735*	−28.871*	1.730*	1.995*	0.032*	−3.871*	−26.592*	1.67E-4*	0.068*	0.065*	−4.472*	1.11E-4*	−0.026*	−0.035*	15,213.451	0.854	< 0.001*

*NA* Not applicable, *NS* Not significant, *SG* Singaporean, *nonSG* non-Singaporean

^a^Refers to analysis of the entire population of hand injuries without the subgroup analysis

^b^17 to 62 years of age

*Statistically significant (*p* < 0.05)

#### Main group analysis

Main group refers to analysis of the entire population of hand injuries without the subgroup analysis. Univariate analysis (Table 3) showed that the correlations were significant (*p* < 0.05) between incidence rate of hand injuries and monthly PMI (R^2^ = 0.084, *p* = 0.008), monthly IPI (R^2^ = 0.053, *p* = 0.035), monthly ElecGenCon (R^2^ = 0.141, *p* = < 0.001), monthly TransSea (R^2^ = 0.121, *p* = 0.001), monthly HealthC (R^2^ = 0.054, *p* = 0.033), quarterly PMI (R^2^ = 0.161, *p* = 0.034), quarterly TransSea (R^2^ = 0.187, *p* = 0.021) and quarterly SGX (R^2^ = 0.001, *p* = < 0.001).

Multiple linear regression (Table 5) showed that the combination of economic variables in the quarterly analysis of AgriLive, AgriFish, PMI, IPI, Gas, CI, CPI, TransAir, TransLand, and F&B gave a combined highest significant (*p* < 0.05) R^2^ value of 0.623, or 62.3%. This means that 62.3% of the number of hand injuries can be explained by these variables in our regression model, in contrast to the R^2^ value of 74.7% from the SARIMA model. For monthly comparison, AgriLive, AgriFish, MI, PMI, IPI, ElecGenCon, Gas, CI, CPI, TransAir, TransSea, TransLand, F&B, SGX, GOR and HealthC were significant (*p* < 0.05) independent variables for the incidence of hand injuries, yielding a R^2^ of 0.225 or 22.5%, in contrast to the R^2^ of 23.8% from the SARIMA model.

##### Formulation of equation

Using the quarterly analysis, an equation to predict the number of hand injuries was derived from the significant independent economic variables (Table 5):

$$Number\ of\ {hand\ injuries}^a=-4.183E-5\ (AgriLive)-0.003\ (AgriFish)+11.640\ (PMI)+2.574\ (IPI)-0.034\ (Gas)+0.908\ (CI)+0.001\ (TransAir)+0.145\ (TransLand)-7.372\ \left(F\&B\right)+2736.439$$

Legend:

^a^Refer to Table 1 for the list of abbreviations.

Variables were smoothed to simple moving average of 3 months.

##### How to use the equation

The equation is used in the following way: To predict the number of hand injuries, using the economic indicator of PMI as an example, keeping all other variables constant, if PMI goes up by 1%, there will be a 11.640 increase in the number of hand injuries.

#### Subgroup analysis

##### Age

In the univariate analysis (Table 4), correlations were significant (*p* < 0.05) between incidence rate of hand injuries for monthly AgriFish (R^2^ = 0.064, *p* = 0.021), monthly MI (R^2^ = 0.092, *p* = 0.005), monthly Gas (R^2^ = 0.136, *p* = 0.001), monthly CI (R^2^ = 0.056, *p* = 0.031), monthly HealthC (R^2^ = 0.050, *p* = 0.040), quarterly AgriLive (R^2^ = 0.314, *p* = 0.002), quarterly MI (R^2^ = 0.186, *p* = 0.022), quarterly Gas (R^2^ = 0.337, *p* = < 0.001), quarterly F&B (R^2^ = 0.353, *p* = < 0.001), quarterly GOR (R^2^ = 0.285, *p* = 0.003) and quarterly HealthC (R^2^ = 0.312, *p* = 0.002).

Multiple linear regression showed that the combination of economic variables in the quarterly analysis gave a combined highest significant (*p* < 0.05) R^2^ value of 0.477, much lower than the R^2^ of 74.7% from SARIMA. Monthly comparison yielded a R^2^ value of 0.275 which is higher than the R^2^ of 23.8% from SARIMA.

#### Resident status

Multiple linear regression (Table 5) for monthly analysis resulted in Resident (Singaporean) status yielding a higher R^2^ (0.332) than SARIMA (R^2^ = 0.238) while Non-resident (Non-Singaporean) status also yielded a higher R^2^ (0.495) than SARIMA. In the quarterly analysis, Resident status had lower R^2^ (0.430) than SARIMA (R^2^ = 0.747), while Non-Resident status had higher R^2^ (0.898) than SARIMA (R^2^ = 0.747).

#### Gender

In the multiple linear regression (Table 5) for monthly analysis, both male (0.409) and female (0.839) subgroups had a higher R^2^ than SARIMA (0.238). In the quarterly analysis, male (0.461) had lower and female (0.854) higher R^2^ than SARIMA (0.747).

## Discussion

While there are several methods for modeling in time series forecasting, including SARIMA and exponential smoothing methods, SARIMA models have shown better performance in predicting road traffic injuries in relation to gender [19]. Advantages of the SARIMA model include autoregressive, moving average and seasonal functions for trend, auto-correlation, smoothing and season [34]. Previously acknowledged disadvantages of the SARIMA model include the requirement of longitudinal data with a large sample size [19]. The number of data points necessary to develop a time series model has been determined to be at least 52 [38] or 80 data points in which 7 years of data is to be collected given the availability of monthly data [39]. With a data collection period of 2012 to 2018, our study has sufficient data points that would allow the SARIMA model to be used. Comparing SARIMA models in the monthly and quarterly analysis, the monthly analysis yielded a low R^2 ^of 0.238 while the quarterly iteration had a higher R^2 ^of 0.747. This suggests that the quarterly SARIMA model is a better predictor of hand injuries.

When comparing for citizenship status, in the quarterly analysis, multiple linear regression for non-Singaporeans suffering from hand injuries yielded a higher R^2^ (0.898) than Singaporeans (0.430). A possible explanation could be the rise in work permits given out in the period of 2012 to 2018. In particular, the total number of work permits issued for semi- and low-skilled jobs in 2014 was 991,300, however in the short span of 6 months in January to June 2015, 993,900 work permits for blue collar jobs (construction workers and factory workers) were issued [40]. Another contributory factor is the large proportion of foreigners that make up ground constructions teams, with figures as high as 90% being reported and a 78% proportion of foreign workforce relative to total employment in construction [41]. The Singapore government has also predicted that two-thirds of Singaporeans will hold white-collar jobs by 2030, up from half the workforce in 2013 [42].

In both construction and fishing industries, regardless of gender, there is a higher incidence of hand injuries. In the monthly comparison, the R^2^ for CI and AgriFish were 0.171 and 0.128 respectively for males; while in females this was 0.531 and 0.138. In the quarterly analysis, the R^2^ values were 0.204 (CI) vs. 0.157 (AgriFish) for males and 0.516 vs. 0.297 for females. This finding is likely attributed to the larger workforce in Singapore’s construction industry as opposed to the fishing industry. In 2013, it was reported that the main source of labour in Singapore’s fishing industry are the fish farm owners and his or her family members. Such a fish farm would employ less than 20 workers, even when seasonal workers are included. Many of these firms are labour-intensive with minimal automation [43].

Since men often work in more dangerous jobs than women and appear to have higher overall injury rates [44], we expected that the multiple linear regression model would fit better for men than women. However, our findings were contrary to this expectation. In both the monthly and quarterly analysis, the female subgroup analysis had a higher R^2^ than males. A previous study amongst aluminum smelter workers found that the injury rate was higher for females compared to males, arriving at the conclusion that women receive less on-the-job safety mentoring from supervisors and coworkers than men [45]. Male workers also tend to have more autonomy and control at work [46, 47], leading to a greater agency in the duration of work that they are exposed to compared to their female counterparts. It has been demonstrated that employees working long hours are more vulnerable to diverse types of occupational health problems [48] including hand injuries. These factors are likely more evident in industrial workforces traditionally dominated by men and could account for female workers having significantly higher risks of all injuries compared to male colleagues [49]. Our findings thus suggest a greater need for occupational workplace precautions and education amongst women to prevent the incidence of hand injuries.

With 17 to 62 being the legal working ages in Singapore [31, 32], we expected that the subgroup analysis for age would yield a higher R^2^ than SARIMA. This hypothesis held true in the monthly multiple linear regression, where R^2^ for the age subgroup yielded a higher R^2^ of 0.275 than SARIMA (0.238). However, this trend was not present in the quarterly analysis with the age subgroup analysis yielding a lower R^2^ of 0.477 than SARIMA (0.747). The rate of accidents and occupational injuries has been reported to be higher among blue-collar compared to white-collar workers [50–52]. The contracts for blue-collar work are relatively short-term relative to white-collar contracts [53], possibly explaining how the monthly analysis is better able to reflect the hand injuries suffered by blue-collar workers than the quarterly analysis.

After controlling for citizenship, we found that manufacturing index (MI) remained a significant dependent variable in the univariate analysis. The R^2^ for MI in the monthly analysis was 0.079, *p* value = 0.010, for Singaporeans and 0.305, *p* value = < 0.001 for non-Singaporeans, while this was 0.148, *p* value = 0.044 vs. 0.508, *p* value = < 0.001 in the quarterly analysis. This suggests that the higher the MI, the higher the rate of hand injuries regardless of nationality. The proportion of the foreign workforce relative to total employment is about 56% in manufacturing [41], indicating an almost equal percentage of Singaporean and non-Singaporean workforce in the manufacturing industry. We also found that TransAir was significant regardless of citizenship. While we were unable to find publicly available figures describing citizenship demographics in Singapore’s air transport cargo industry, we know that advances in automation in cargo operations industry could lead to a decrease in hand injuries. For example, previous cargo operation assistants previously had to manually sort and lift mail bags, which can be as heavy as 35 kg. However, since the introduction of automation in the form of automated tilt-tray sortation systems for intelligent processing capabilities and assisted loading devices for lifting mail bags, workers have undergone training to operate automated systems as technical specialists [54].

Singapore’s labour force has fluctuated across the years. The Report on Labour Force in Singapore by the Ministry of Manpower details that between 1994 to 1996, the percentage change year on year was 4.5 to 5.04% to 15.75% respectively [11]; and from 2012 to 2018, the percentage change was 3.71 to 2.38% to 2.47 to 2.21% to 1.69% to − 0.43% to 0.51% respectively [55]. In 2018, Singapore’s Ministry of Manpower’s reported that the total labour force was 3,675,600, with the employment rate being 97.3% (3,575,300) [11]. With a high employment rate of 97.3%, economic indicators such as the Straits Times Index (STI) and Gross Domestic Product (GDP) performed well for that year, with the 2018 economy expanding by 1.9% from 2017 [56].

In Singapore, hand injuries make up a significant portion of acute injuries [57], many of which are sustained in the industrial workplace. In 2020, there were 46 out of 463 (9.34%) cases where workers lost their hands or fingers in amputation accidents [58, 59]. The number of hand injuries would fluctuate with fluctuations in the total number of labour force in a country.

PMI is a measure of the prevailing direction of economic trends in manufacturing, summarising whether market conditions from the perspectives of managers are expanding, staying the same or contracting [60–62]. CI indicates the level of economic activity in the Construction sector [63]. Labour Productivity Change indicates whether output is increasing or decreasing per worker [64, 65]. Greater productivity describes being able to do more in the same amount of time, this in turn frees up resources to be used elsewhere.

Qualitatively, for example, as Purchasing Manager Index (PMI) is based on a monthly survey of supply chain managers across various industries, if these managers feel they expect a higher demand from customers for their goods, they will increase their orders. This provides a favorable outlook to the overall economic activity. Correspondingly, based on our equation, the number of hand injuries would increase. In like manner, if Labour Productivity Change for Manufacturing increases, which means that the hourly manufacturing economic output produced by an hour of labour rises, then there will also be a reciprocal increase in the number of hand injuries.

### Strengths

Regression models could be of value in resource utilisation by targeting the individuals in occupations or workplaces that need the most intervention [26]. Thus, prediction of hand injuries can provide a useful tool for occupational health safety policymakers by simulating changes in economic variables when applying new workplace or manpower interventions and regulations in the future.

To our knowledge, this is the first study to analyse the correlation of economic factors against the number of hand injuries. In our data, there is a relatively high proportion of foreign patients (41.21%) that undergo hand surgeries. As of 2018, the total foreign workforce in Singapore stood at 1,386,000 (24.58% of the entire population). However, they are usually under-represented in studies. In this study, we present a large cohort of foreign patients compared to the national proportion.

The clinical implications and findings of this study may allow us to forecast the clinical resources required to treat hand injuries from the construction, manufacturing and transport industries in relation to economic indicators. 

### Limitations

We identified a few limitations in our study. Firstly, we used the incidence rate for hand surgeries as a proxy for the incidence rate for hand injuries. These hand surgeries included both elective and emergency operations and therefore may not be reflective of patients who presented with hand injuries but did not undergo hand surgeries. However, our large sample size of 20,764 patients is likely to account for exceptions pertaining to these cases. Hand injuries are typically emergency injuries such as lacerations and crush trauma; but our data combines both. Fortunately, elective operations are not known to fluctuate much. For example, trigger finger operations are not known to fluctuate from month to month [66–68]. Since we have found in our results that there is a significant correlation between the incidence of hand injuries and economic indicators, an extrapolation between two time points would effectively nullify the electives, leaving the emergencies to account for the cause of fluctuations.

Secondly, our tertiary institution covers referrals from the south and western regions of Singapore where a higher proportion of construction and manufacturing industries are located. Hence, there may be a higher proportion of hand injuries that may present to our institution as opposed to other local tertiary institutions. However, this allows us to have an increased sensitivity to the fluctuations in hand surgeries done emergently in response to fluctuations in the labour force.

Lastly, some of the economic markers used in our study were not reported in a timely fashion. For example, PMI is reported monthly, because companies use it to purchase equipment but others such as CI were published 1 year later. Therefore, the reporting time lag of the economic variables may dampen the usage of our equation as a timely method of forecasting.

## Data Availability

Data may be made available upon reasonable request.
